# Leaf surface characteristics affect the deposition and distribution of droplets in rice (*Oryza sativa* L.)

**DOI:** 10.1038/s41598-021-97061-5

**Published:** 2021-09-08

**Authors:** Guangmei Ji, Huizhe Chen, Yuping Zhang, Jing Xiang, Yaliang Wang, Zhigang Wang, Defeng Zhu, Yikai Zhang

**Affiliations:** 1 College of Agronomy, Jiangxi Agricultural University, Nanchang, Jiangxi 330045 People’s Republic of China; 2 State Key Laboratory of Rice Biology, China National Rice Research Institute, Hangzhou, Zhejiang 310006 People’s Republic of China; 3 Guizhou Rice Research Institute, Guiyang, Guizhou 550009 People’s Republic of China

**Keywords:** Physiology, Plant sciences

## Abstract

We studied the effects of leaf surface characteristics on canopy droplet behaviour using two rice cultivars with similar leaf shapes but significantly different leaf surface characteristics: Jia58 (glabrous rice; smooth leaf surface and no burrs) and Yongyou12 (hairy-leaved rice; rough leaf surface covered with burrs). The plants were subjected to spray tests with different spray pressures and nozzle apertures. The results showed that the deposition amount per unit leaf area was significantly higher in the Yongyou12 canopy than in the Jia58 canopy. The diameter, volume median diameter, number median diameter, and coverage of droplets were significantly higher in Yongyou12 than in Jia58, while the coverage density of droplets was significantly lower. The proportion of small droplets of Jia58 is higher than that of Yongyou12. Thus, a larger amount of large-sized droplets could retain on the leaf surface of hairy-leaved rice, and a larger number of small-sized droplets were retained on the leaf surface of glabrous rice. Smaller pressure and larger flow nozzle were conducive to the retention of the Jia58, while Yongyou12 required larger pressure and larger flow nozzles. Ultrastructural analyses revealed that the leaf surface of glabrous rice had no trichomes and more wax than hairy-leaved rice, and the critical surface tension was lower, resulting in the retention of mainly small droplets on its leaf surface and a lower deposition amount. Therefore, in order to increase the deposition of pesticide droplets on the leaf surface in production, glabrous rice should choose nozzles with smaller spray pressure and large flow rate.

## Introduction

Rice is one of the most important food crops in China, and its planting area and total production are important for China’s food production and food security^1^. However, diseases, insects, grass weeds, and other biological factors are affecting the stable and high yield of rice in China^2,3^. The annual yield loss of rice in China due to pests and diseases is about 4 million tons^4,5^. Chemical pesticides are the main pest control method used in rice production, and the area of pesticide use in China amounts to more than 167 million hectares per year^6,7^. Due to sub-optimal application technologies, a large amount of pesticide is not delivered to the right place or is unevenly applied. This reduces its effectiveness to control pests, and results in the waste of pesticides and pollution of soil and water. In China, the annual pesticide-contaminated area is 13.3 million hectares, accounting for more than one-seventh of the national arable land^8^. Therefore, improving application technologies and increasing the utilization rate of pesticides is a priority for China’s food production. In addition to the spraying machinery, application methods, and pesticide composition, the effective use of pesticides also depends on the leaf morphology of crop plants^9^. Diseases and insects occur at different times and in different parts of rice plants^10^. Because plant height and leaf morphology differ among rice varieties, pesticides need to be applied considering these characteristics and the site where diseases and insects occur^11^. It has been shown that the droplet deposition amount is significantly affected by leaf surface properties and leaf inclination angle^12^. Nozzle type, size and pressure also affect the amount of deposition on crop leaves^13^. The amount of pesticide deposited on the leaf is closely related to the effectiveness of pest and disease control. However, no previous studies have focused on differences in the deposition and distribution of pesticides on the leaf among varieties with different leaf surface characteristics. In the present study, we selected glabrous rice^14^ (a dominant rice species with smooth leaves, stem sheaths, and rice grains) and hairy-leaved rice with a similar leaf shape but significantly different leaf surface characteristics, and applied a liquid with different spray pressure and nozzle aperture. This allowed us to compare the deposition of spray droplets between these two varieties, and to study the behaviour of canopy droplets on rice plants with different leaf surface characteristics. Ultimately, our results provide a basis for the improvement of spraying technology to reduce the amount of pesticide applied and improve its utilization.

## Results

### Droplet deposition on leaf canopy

**Figure 1:**
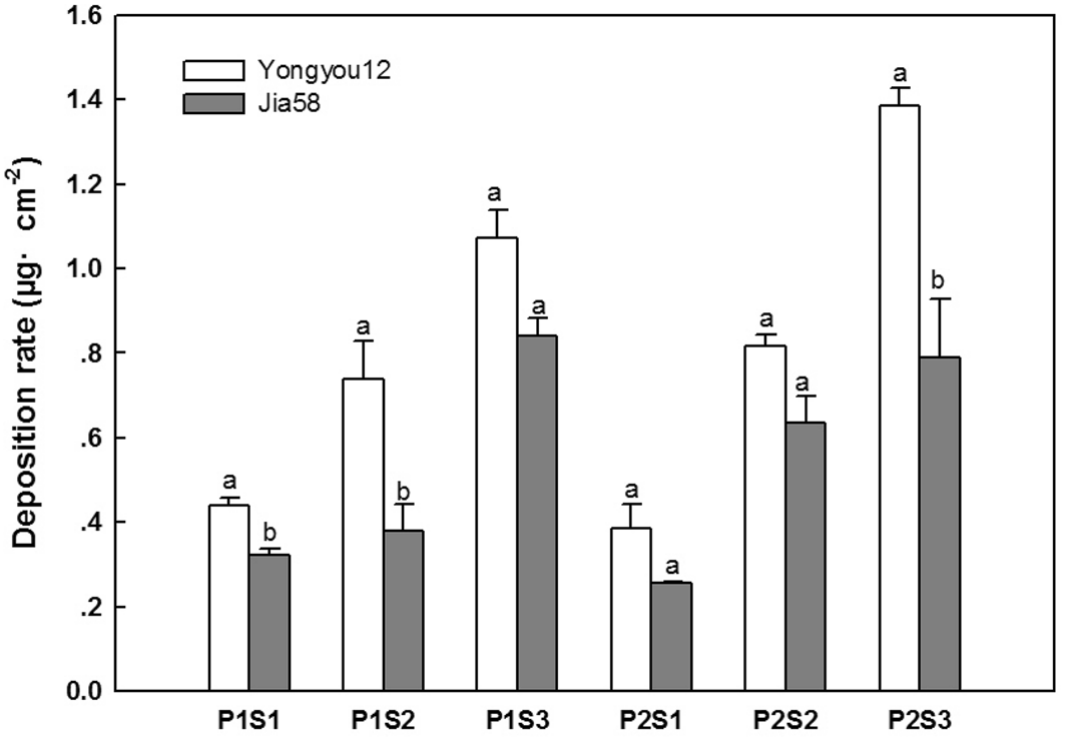
Droplets deposition on the canopy leaves of two rice varieties. The different letters means significant difference (*P* ≤ 0.01) by t-tests.

Spray pressure and nozzle aperture are the key factors affecting droplet deposition on the canopy of rice, and the deposition pattern of droplets on the canopy is closely related to the leaf surface characteristics. Under the same spraying conditions, the deposition amount per unit leaf area was significantly higher in Yongyou12 with hairy leaf than in Jia58 with hairless leaf (Table 1, Fig. 1). The deposition per unit area of Yongyou12 was 0.81 μg cm^−2^, while that of Jia58 was only 0.54 μg cm^−2^. With the increase of spray pressure, the deposition amount of Yongyou12 and Jia58 increased by 0.11 μg cm^−2^ and 0.05 μg cm^−2^, respectively, and the difference was not significant. Under the same spray pressure, the deposition of canopy droplets in both rice varieties increased significantly with increasing nozzle aperture. As the nozzle becomes larger, the deposition volume of Yongyou12 increased by 0.36 μg cm^−2^ and 0.45 μg cm^−2^, respectively, while the deposition volume of Jia58 increased by 0.22 μg cm^−2^ and 0.31 μg cm^−2^ respectively. Comparison of deposition under different conditions revealed that Yongyou12 received 1.39 μg cm^−2^ canopy droplets per unit area under the optimal deposition condition P2S3, while Jia58 received 0.84 μg cm^−2^ of canopy droplets per unit area under the optimal deposition condition P1S3. Therefore, in the actual spraying operation, glabrous rice should be sprayed with a nozzle with a lower spray pressure and a larger flow rate, which is more conducive to the deposition of pesticide drops on the surface of the blade.

**Table 1:** Results of ANOVA in the rice deposition and distribution of droplets.

Sources of variation	Deposition rate	Droplet coverage density	Droplet coverage rate	Droplet diameter	Number middle diameter	Volume middle diameter
Genotype (G)	10.09**	864.11***	237.35***	122.65***	97.77***	38.36***
Spray pressure (P)	80.19***	33.54***	52.51***	19.18***	1.76NS	0.02NS
Nozzle size (S)	1021.79***	3.21NS	99.86***	8.54**	4.12*	7.38**
G × P	0.56^NS^	189.98***	4.81*	14.14***	1.41^NS^	0.009^NS^
G × S	0.34^NS^	27.48***	36.29***	5.49*	1.86^NS^	0.61^NS^
P × S	99.23***	129.84***	21.55***	4.51*	1.46^NS^	2.59^NS^
G × P × S	0.24^NS^	4.11*	26.14***	14.62***	7.96**	4.36*

*NS* not significant.

*Denotes significance at the P ≤ 0.05 level.

**Denotes significance at the P ≤ 0.01 level.

***Denotes significance at the P ≤ 0.001 level.

### Droplet distribution in leaf canopy

**Figure 2:**
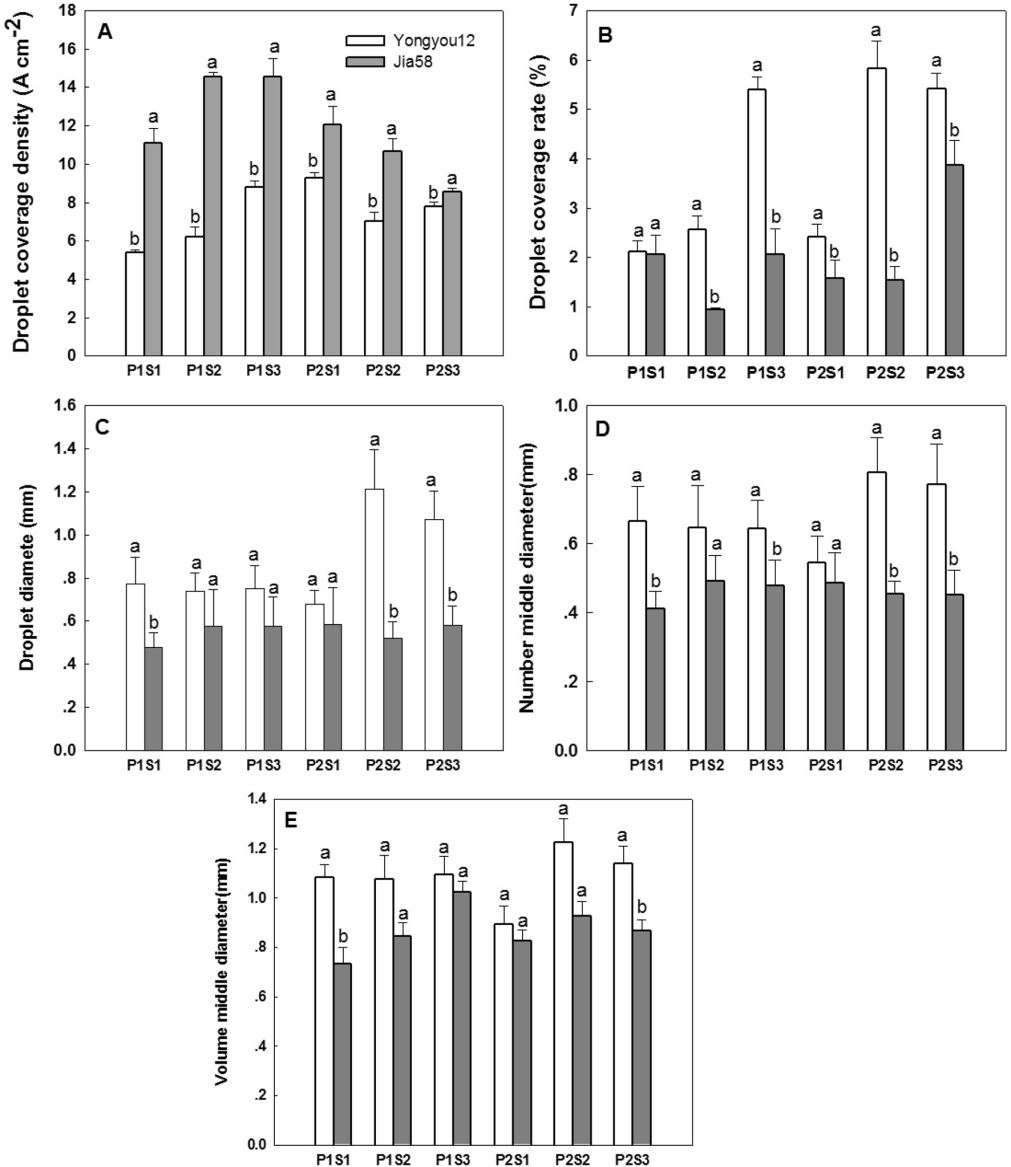
Distribution of droplets on the canopy leaves of two rice varieties. The different letters means significant difference (*P* ≤ 0.01) by t-tests.

The droplet coverage density was significantly lower in Yongyou12 than in Jia58 (Table 1, Fig. 2A). For Yongyou12, the larger spray pressure increased the droplet coverage density, while the larger spray pressure of Jia58 is not conducive to the droplet coverage. Under low spray pressure, the higher nozzle flow rate promoted the increase in the leaf droplet coverage density of the two genotypes of rice, while the higher nozzle flow rate reduced the leaf droplet coverage density of the two varieties with high spray pressure. However, droplet coverage was higher in Yongyou12 than in Jia58 (Table 1, Fig. 2B). Similarly, the droplet diameters (Fig. 2C), the number median diameter (Fig. 2D) and the volume median diameter (Fig. 2E) were larger for Yongyou12 than for Jia58. For both varieties, the volume median diameter of droplets was much larger than the droplet diameters and the number median diameter of droplets. The three diameters of Yongyou12 showed an increasing trend with increased spraying pressure and nozzle aperture, but those of Jia58 were not significant. Therefore, a larger amount of larger-sized droplets could retained on the leaf surface of Yongyou12, but a larger number of smaller-sized droplets were retained on the leaf surface of Jia58.

### Droplet size in leaf canopy

According to the average droplet diameter, the droplets formed on the leaf canopy surface after spraying can be classified into different groups. This clarifies the dynamics and variability of droplet distribution of different particle sizes, and is the key to understanding the uniformity of droplet distribution in the canopy. As shown in Table 2, the proportion of small droplets (D1) of Yongyou12 was lower than Jia58 while the proportion of large droplets (D3) of Yongyou12 was higher than Jia58, and the droplet uniformity (D2) was Yongyou12 higher than Jia58. For Yongyou12, as the spray pressure increased, the number of droplets in the D1 and D3 groups decreased while the number of droplets in the D2 groups increased, i.e., the droplet uniformity increased. For Jia58, as the spray pressure increased, the droplet size distribution law was opposite to that of Yongyou12. The nozzle aperture did not significantly affect the pattern of droplet uniformity. Yongyou12 had the highest droplet uniformity under P2S3 treatment, while Jia58 had the highest droplet uniformity under P1S1 treatment. Therefore, compared with hairy rice, the leaf surface of glabrous rice was easier to retain small droplets, and the droplet uniformity was lower.

**Table 2:** Rice canopy different size distribution of droplets.

Treatment	D1 (%)		D2 (%)		D3 (%)	
	Yongyou12	Jia58	Yongyou12	Jia58	Yongyou12	Jia58
P1S1	41.53	46.66	48.20	46.79	10.27	6.55
P1S2	44.96	51.95	42.78	40.02	12.25	8.03
P1S3	43.02	48.51	45.91	43.67	11.08	7.82
P2S1	39.47	55.18	52.44	35.60	8.09	9.22
P2S2	40.10	49.35	50.77	44.03	9.14	6.61
P2S3	38.25	52.59	53.93	40.05	7.82	7.35

The average diameter of all droplets is D, grouped by 0–80%D, 80–160%D, > 160%D (numbers are D1, D2, D3).

### Ultrastructure of rice leaf surface

**Figure 3:**
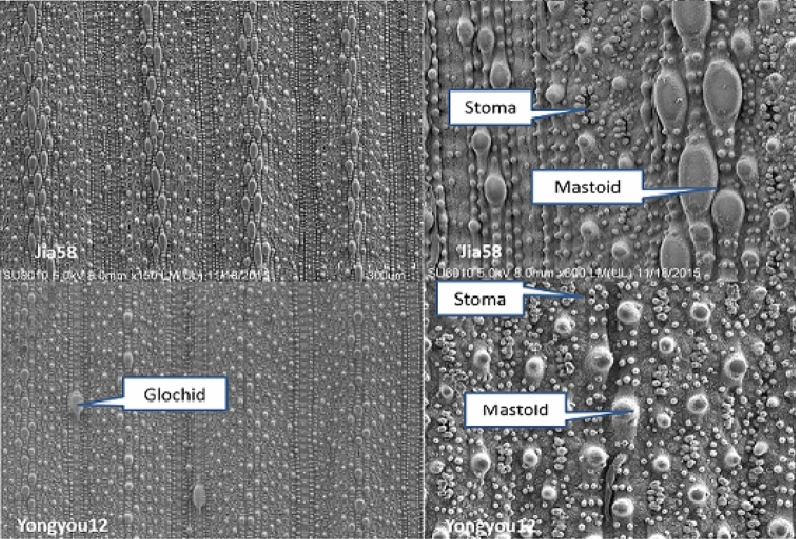
Scanning electron microscope(SEM)photos of two rice varieties. 100 μm and 50 μm are mage scale, is under 3000 times and 6000 times the figure.

The leaf surface structure varies greatly among different types of rice varieties. As shown in Fig. 3, the leaf surface of Yongyou12 is densely covered with papillae, stomata, and other microstructures, and the papillae have a rough surface, are mainly spherical, and are scattered. The leaf surface of Jia58 is also densely covered with papillae, stomata, and other microstructures, and the surface of papillae is smoother. There are ellipsoidal and spherical papillae, with the ellipsoidal papillae being larger and densely arranged along the leaf veins, and the spherical papillae being smaller and scattered. Analyses of leaf surface characteristics showed that the papillae density and leaf wax content were significantly lower in Yongyou12 than in Jia58, while the stomatal density, contact angle, and critical surface tension were higher in Yongyou12 than in Jia58 (Table 3). Glochids were present on the leaf surface of Yongyou12, but not on Jia58. The leaf surface of Yongyou12 was favourable for the deposition and retention of large droplets on the leaf surface during spraying, while the leaf surface of Jia58 were not. Our results on droplet deposition and distribution were also consistent with this.

**Table 3:** Characteristics of leaf surface in two rice varieties.

Blade surface features	Unit	Cultivar	
		Yongyou12	Jia58
Papillae density	10^3^ mm^−2^	6.5 ± 0.4b	8.5 ± 0.4a
Stoma density	A mm^−2^	440 ± 8.0a	390 ± 12.2b
Glochid density	A mm^−2^	6.7 ± 0.3a	0.0b
Blade wax content	Mg cm^−2^	1.99 ± 0.1b	6.04 ± 0.3a
Critical surface tension	mN m^−1^	38.8 ± 8.8a	37.9 ± 7.9b

The different letters means significant difference (*P* ≤ 0.01) by t-tests.

## Discussion

The microstructure, wax, stomata, and leaf surface attachment hairs and thorns on the surface of plant leaves are more important influencing factors in the process of pesticide droplet deposition^15^. The electron microscope showed that rice leaves were covered with papillary protrusions (papillae) and coated with wax, which specific structure led to strong hydrophobicity of rice leaves^16^. In this study, papillae density of Jia58 was significantly higher compared with Yongyou12. The wax on the surface of the plant greatly affected the droplet wetting and other behaviors, and the C29, C33, and C35 long-chain hydrocarbons in the surface wax of rice leaves were more than 90%, showing strong hydrophobicity^17^. In the study, the leaf surface structure of Jia58 differed greatly from that of Yongyou12, and the content of waxes was significantly higher in Jia58 than in Yongyou12. The epicuticular wax content in the leaves of cabbage (*Brassica oleracea*) was particularly high, and when spraying in a large volume, the liquid with higher surface tension would easily rebound on the leaves or roll down along the petiole, resulting in lower pesticide utilization^18^. The critical surface tension of glabrous rice was significantly lower than that of hairy-leaved rice, which indicated that the pesticides were not easy to stay on the surface of the leaves of glabrous rice. Finally, the deposition amount per unit leaf area was significantly lower on the glabrous rice Jia58 than on the hairy-leaved rice Yongyou12.

The burrs and thorn attachments on the leaves surface of plant significantly affected the deposition and adhesion behavior of pesticide droplets^5,19,20^. The glochid easily pierced the surface of the droplet, making the contact angle of the droplet on the plant leaf surface smaller^21^. The hairy-leaved rice Yongyou12 had much glochids on the leaf surface, which increased the leaf surface coverage and promoted the deposition of droplets on the leaf surface (Fig. 1). Song et al. (2013) found that droplets deposited more in silicified bands of rice leaves and mainly on glochids of leaves^22^. On the surface of rice leaves, there are papillae on stomatal band, and there are papillae and glochids on silicified bolt band. The size of glochid was 30–40 times of papillae. The ability of burrs and silicified bolt zone on rice leaf surface to adsorb foreign droplets was significantly stronger than that in other regions, and the droplet attachment accounted for more than 80%^23^. The number of papilla density and wax content were significantly higher in Jia58 than in Yongyou12, and the glochid density and stoma density of Yongyou12 were lower than those of Jia58, which resulted in significantly lower leaf surface deposition on glabrous rice than on hairy-leaved rice under the same spraying conditions.

The droplet size, droplet volume median diameter, and droplet number median diameter strongly affected droplet deposition^20^. Differences in rice leaf surface properties could lead to different diffusion, aggregation, and even bouncing of droplets on the leaf surface, all of which affected droplet distribution^5,19,20^. In this study, the droplet coverage density was significantly higher on Jia58 than on Yongyou12; the former had smaller droplet diameter, droplet volume median diameter, and droplet number median diameter, and the deposited droplets were smaller but more abundant than those deposited on the leaves of hairy-leaved rice. The critical surface tension of glabrous rice was relatively lower, which surface can be attached to the small particle size droplets with small dynamic surface tension. In pesticide spraying, each droplet had an effective killing radius for pest control, and a certain number of droplets accumulated per unit area could achieve good control effects^2,11^. Compared with hairy-leaved rice, glabrous rice accumulated more small-sized droplets, which reduced the amount of spray solution required for adequate coverage. The leaf surface of hairy-leaved rice was rough, with glochid, which had a larger surface tension and lower wax content. The droplets deposited on it were larger than those deposited on the leaf surface of glabrous rice. Larger pressure and larger flow nozzle were beneficial to the deposition of hairy-leaved rice, while smaller pressure and larger flow nozzle were conducive to the retention of glabrous rice.

Compared with hairy-leaved rice, leaves of glabrous rice lacked trichomes, had a higher wax content and lower critical surface tension. When pesticide was sprayed onto the leaf surface of glabrous rice, the deposition amount was low and the droplets were small with a good coverage density. This means that a lower dosage of pesticides and spray pressures was required for effective pest control in glabrous rice than in hairy-leaved rice. Therefore, when spraying in the field, full consideration should be given to the leaf surface properties of glabrous rice, and the use of lower spray pressure and large flow nozzles could better improve pest control by ensuring adequate, but not excessive, pesticide delivery.

## Materials and methods

### Experiment design and plant materials

A pot experiment was conducted at the Experimental Farm, China National Rice Research Institute, Hangzhou, China in 2015. For all experiments on the rice plants, we confirm that all methods were carried out in accordance with relevant guidelines and regulations. Two cultivars were chosen with different leaf surface characteristics. Jia58 with upright leaves, smooth and hairless and Yongyou12 with upright leaves and hairy leaves were used in the experiment^14^, which were obtained from the Jiaxing Academy of Agricultural Sciences, Zhejiang, China and Ningbo Seed Co., Ltd., China, respectively. The pot experiment was adopted, and the pot size was 20 cm × 18 cm × 25 cm, and each pot contained 8 kg of soil. The test soil was paddy soil (pH5.93, organic matter 28.3 g kg^−1^, total nitrogen 1.6 g kg^−1^, available nitrogen 128.2 mg kg^−1^, available phosphorus 44.8 mg kg^−1^, available potassium 130 mg kg^−1^). The nutrient elements were adding at the following proportions in each pot: 200 mg kg^−1^ N (as urea), P 150 mg kg^−1^ (as Superphosphate), 150 mg kg^−1^ K (as KCl). Irrigation was carried out in accordance with conventional high-yield cultivation methods, intermittent irrigation was used to regularly control the occurrence of pests and diseases, and rice grew normally.

According to Massinon et al. (2014) with minor modifications, the spray device used in this study is a walking spray tower designed and assembled by the China National Rice Research Institute^24^. It included pressure gauges, flow meters, pressure pumps, spray rods, nozzles, etc. The motor drives a uniform speed to travel. The nozzle type, walking speed, spray pressure and spray flow could be adjusted according to the treatment requirements. Two spray pressure treatments (P1, P2) were set in this study. The nozzle type (S1, S2, S3) used in this study is a standard fan nozzle with a spray fan angle of 110°. A total of 6 treatments were set, namely P1S1, P1S2, P1S3, P2S1, P2S2, P2S3 listed in Table 4, and each treatment selected the same population traits and four replicates. The spray agent was a mixed solution of indicator ponceau-G (mass concentration of 1 g L^−1^) and surfactant NP-10 (mass concentration of 150 mg L^−1^) for spray operation. In the rice heading stage, top-pressure spray is used, and the spray nozzle moves parallel from the top of the potted rice, 50 cm away from the top of the rice. The spray tower kept walking at a constant speed with a speed of 2 m s^−1^. After spraying, single leaves were randomly selected and the image collection device (Sony a7sIII, Sony Corporation, Japan) was used to collect instantaneous images of droplets on the leaf, and 16 single leaves were selected for each process. After the spray liquid was deposited on the surface of the blade and air-dried, separate treatments to collect and sample.

**Table 4:** The treatment selected in the spray test and its corresponding spray pressure and liquid flow.

Treatment	Spray pressure (MPa)	Liquid flow (L min^−1^)
P1S1	0.1	1.09
P1S2	0.1	1.28
P1S3	0.1	1.62
P2S1	0.2	1.68
P2S2	0.2	1.86
P2S3	0.2	2.14

### Methods of measurements

#### Ultrastructure of rice leaf surface

Two freshly fallen leaves at the beginning of the heading stage of the two varieties were selected and fixed with electron microscope fixative, and placed in a refrigerator at 4℃ for more than 4 h. Rinse 3 times with 0.1 mol L^−1^ PBS with copper mesh for 15 min each time, then fix with 1% osmic acid-0.1 mol L^−1^ PBS for 30 min, then rinse 3 times with 0.1 mol L^−1^ PBS for 15 min each time. Dehydrate in 50–70–80–90–95–100–100% (v/v) alcohol successively, 15 min each time. Add isoamyl acetate, completely immerse the leaves, let it stand for 10–20 min, and move it into a critical point dryer for drying. Paste the blade or copper mesh on the specimen table with conductive glue (the observation side is facing up), and use a vacuum coating instrument to coat the film (gold film). The sample is placed in a scanning electron microscope for observation, and images are collected for analysis^25^.

#### Critical surface tension

The contact angle (θ) of the liquid with the same volume and different surface tension on the surface of the rice leaf at the heading stage was measured with a contact angle measuring instrument. A straight line can be obtained by plotting the cosine value cosθ of the contact angle against the surface tension value of the liquid. Extend the straight line to cosθ = 1, the corresponding liquid surface tension value is the critical surface tension of the rice leaf^26^.

#### Leaf epidermis wax

Weigh 2 g of fresh mature leaves, calculate their surface area, and immediately place them in 30 mL of 60 °C chloroform, and take them out immediately after 30 s. After the extract is naturally volatilized, weigh the wax mass and calculate the wax content (ug cm^−2^) = wax mass (ug)/wax surface area (cm^2^)^27^.

#### Droplets deposition

After the spray test, the collected leaves were washed with distilled water and fixed to volume^28^. The absorbance was measured at 510 nm. Weigh 1 g of Ponceau-G and 150 mg of NP-10, dissolve them with distilled water and transfer them to a 1 L volumetric flask to make a mixed mother liquor, respectively, to prepare a mixed solution of different mass concentration gradients of Ponceau-G. Measure the absorbance of each concentration solution at 510 nm, and draw a standard curve, the equation is: y = 18.226x − 0.7456 (R^2^ = 0.9994). According to the standard curve equation and the leaf area, the amount of deposition per unit area of the leaf was calculated.

#### Leaf droplet group analysis

Leaf area from the digital images collected in the spray test using LA-S plant image analysis system (Wseen detection technology Co., Ltd., Hangzhou, China).The number of droplets and droplet area and leaf area from the digital images collected in the spray test using SC-G automatic seed test analysis system (Wseen detection technology Co., Ltd., Hangzhou, China)^29^. The droplet coverage density is the number of droplets deposited on a unit area. The droplet coverage is the ratio of the deposited area on the rice leaf to the leaf area. The droplet diameter is the average diameter of the droplets on the blade. The number median diameter (NMD) refers to the diameter of the droplets arranged on the blade from small to large, and the diameter of the droplets when the number of droplets is accumulated in order from small to large to reach half of the total number of droplets. The volume median diameter (VMD) is the droplet diameter when the volume of the droplets on the blade is added up to half of the total volume of all the droplets in order from small to large^30^.

#### Droplet size analysis

After spraying, the droplets of the rice canopy of each variety of treatment respectively constitute a droplet group*.* In the droplet group, the average diameter of all droplets is D, grouped by 0–80%D, 80–160%D, > 160%D (numbers are D1, D2, D3), analyze the distribution of droplets of different sizes in the rice canopy^30^.

### Statistical analyses

Statistical analyses were performed by one-way analyses of variance using a general linear model using SPSS 21.0 software. Data are presented as means and standard errors below. Statistically significant differences (P ≤ 0.05) between averages were identified by performing t-tests.

## Data Availability

All data generated or analyzed during this study are included in the article.
